# Hospital admissions for skin infections among Western Australian children and adolescents from 1996 to 2012

**DOI:** 10.1371/journal.pone.0188803

**Published:** 2017-11-30

**Authors:** Tasnim Abdalla, David Hendrickx, Parveen Fathima, Roz Walker, Christopher C. Blyth, Jonathan R. Carapetis, Asha C. Bowen, Hannah C. Moore

**Affiliations:** 1 Wesfarmers Centre for Vaccines and Infectious Diseases, Telethon Kids Institute, The University of Western Australia, Perth, Western Australia, Australia; 2 Centre for Child Health Research, University of Western Australia, Perth, Western Australia, Australia; 3 NHMRC Centre for Research Excellence in Aboriginal Health and Wellbeing, Telethon Kids Institute, University of Western Australia, Perth, Western Australia, Australia; 4 School of Medicine, University of Western Australia, Perth, Western Australia, Australia; 5 Department of Infectious Diseases, Princess Margaret Hospital for Children, Perth, Western Australia, Australia; 6 Menzies School of Health Research, Charles Darwin University, Darwin, Northern Territory, Australia; Kliniken der Stadt Köln gGmbH, GERMANY

## Abstract

The objective of this study was to describe the occurrence of skin infection associated hospitalizations in children born in Western Australia (WA). We conducted a retrospective cohort study of all children born in WA between 1996 and 2012 (n = 469,589). Of these, 31,348 (6.7%) were Aboriginal and 240,237 (51.2%) were boys. We report the annual age-specific hospital admission rates by geographical location and diagnostic category. We applied log-linear regression modelling to analyse changes in temporal trends of hospitalizations. Hospitalization rates for skin infections in Aboriginal children (31.7/1000 child-years; 95% confidence interval [CI] 31.0–32.4) were 15.0 times higher (95% CI 14.5–15.5; P<0.001) than those of non-Aboriginal children (2.1/1000 child-years; 95% CI 2.0–2.1). Most admissions in Aboriginal children were due to abscess, cellulitis and scabies (84.3%), while impetigo and pyoderma were the predominant causes in non-Aboriginal children (97.7%). Admissions declined with age, with the highest rates for all skin infections observed in infants. Admissions increased with remoteness. Multiple admissions were more common in Aboriginal children. Excess admissions in Aboriginal children were observed during the wet season in the Kimberley and during summer in metropolitan areas. Our study findings show that skin infections are a significant cause of severe disease, requiring hospitalization in Western Australian children, with Aboriginal children at a particularly high risk. Improved community-level prevention of skin infections and the provision of effective primary care are crucial in reducing the burden of skin infection associated hospitalizations. The contribution of sociodemographic and environmental risk factors warrant further investigation.

## Introduction

Skin and soft tissue infections have an important public health impact globally [1–3]. Although rarely fatal, skin conditions such as impetigo, scabies and fungal infections are among the most prevalent diseases in the world and contribute substantially to the global burden of disease [1]. Over 162 million children living in low and low-middle income countries are affected by impetigo at any one time (most frequently caused by *Staphylococcus aureus* or *Streptococcus pyogenes*) [2], while the global prevalence of scabies (caused by the mite *Sarcoptes scabiei*) has been estimated at 100 million cases, with the highest burden found in children living in tropical climates [4]. In Australia, high rates of skin infections have been documented in children of Aboriginal and/or Torres Strait Islander descent (herein referred to as Aboriginal) living in remote Indigenous communities [2,5], where prevalence rates as high as 50% for scabies and 90% for impetigo have been documented in some areas [2,6]. However, most published studies are from remote, tropical communities of the Northern Territory (NT) [5]. The few studies available for Western Australia (WA) are consistent with the NT findings [5,7,8]. Published data on the burden of skin infections in children elsewhere in Australia are minimal, and hospitalization data are limited [9–11]. Skin infections are generally considered a primary health issue [2] and their potential impact on hospital admissions has not previously been documented comprehensively. We aimed to describe the hospital admission profile for skin infections in a cohort of children born in WA between 1996 and 2012.

## Methods

### Population and setting

**Fig 1:**
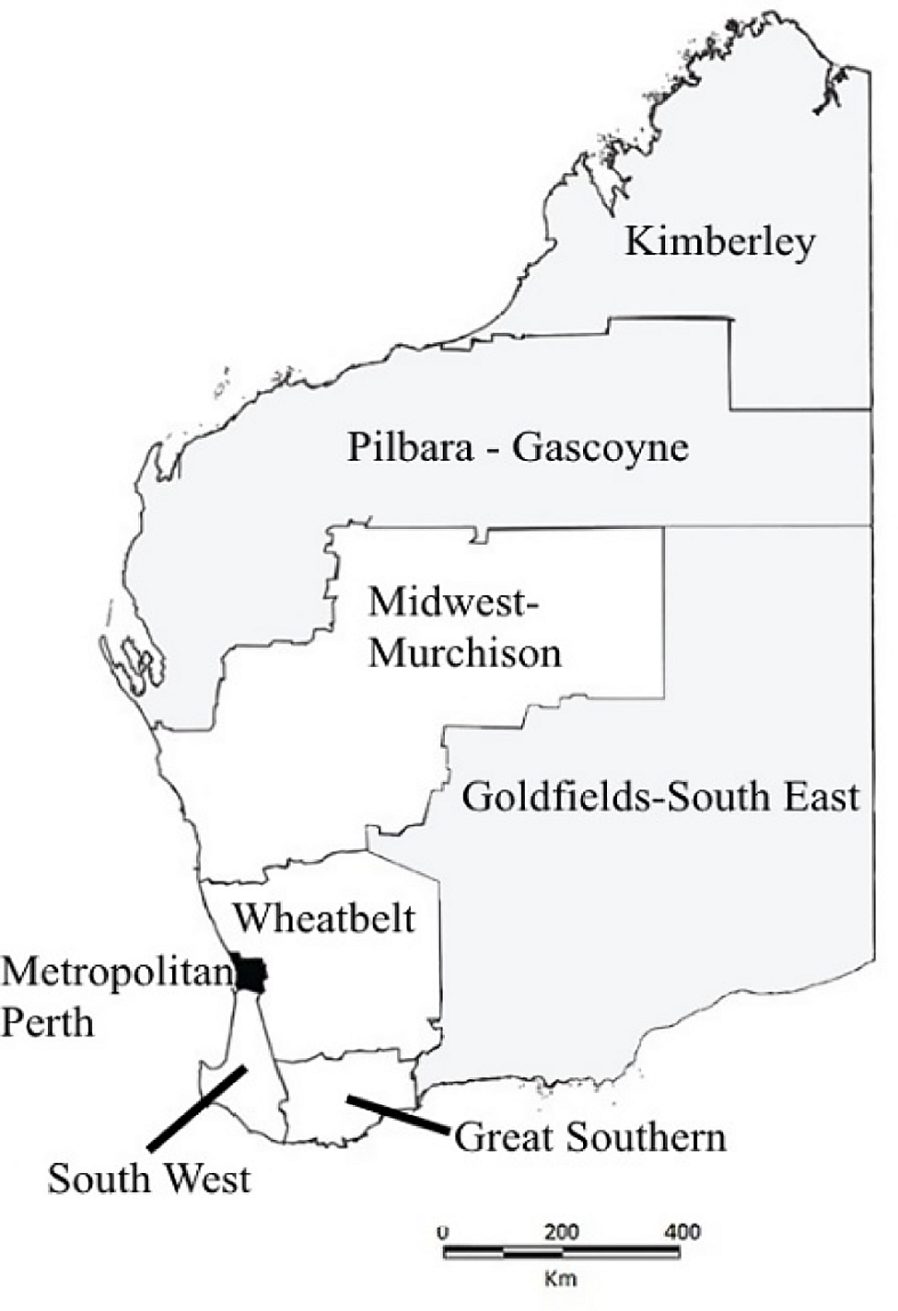
Map of Western Australia. Shows metropolitan (black), rural (white) and remote (grey) areas.

WA extends over approximately 2.6 million square kilometers, spanning the entire western third of Australia. In 2011, WA had a population of 2.4 million people, 3.7% of whom identify as Indigenous, predominantly Aboriginal [12]. The state is divided into health administrative regions, comprising Perth metropolitan (North and South), rural (Midwest-Murchison, Wheatbelt, Great Southern, South West) and remote regions (Pilbara, Kimberley, Goldfields) (Fig 1). Most Western Australians (73.5%) live in the Perth metropolitan area, with the remainder living in regional and remote regions [13]. Relatively higher proportions of Aboriginal people reside in regional, remote and very remote regions (65.2%) in comparison to non-Aboriginal people (28.7%) [14]. In 2012, children and young people aged 0–17 years comprised 22.9% of the state’s population, 73% of which were living in metropolitan areas, 17% in regional areas and 10% in remote regions [15]. The climate in WA varies throughout the state: a warm temperate climate in metropolitan Perth, the south-west and Great Southern areas; dry climate in the Goldfields, Midwest-Murchison and Wheatbelt; and warm, humid conditions in the sub-tropical and tropical northern regions of the Pilbara and the Kimberley [16].

### Study design and data sources

We conducted a retrospective population-based cohort study of all live births in WA between 1996 and 2012, using de-identified probabilistically linked population-based data derived from the WA Data Linkage System[17–19]. Data were extracted from Birth and Death registrations, Midwives Notification System, and the Hospital Morbidity Data Collection (HMDC). The HMDC contains all inpatient separation in public and private hospitals in WA. All admission records contain clinically coded principal and additional diagnoses and procedures. Skin infection diagnosis codes were identified using the Australian version of the International Classification of Diseases 9th revision, Clinical Modification (ICD-9-CM) and 10^th^ revision, Australian Modification (ICD-10-AM). The diagnosis codes were selected and categorized into scabies, impetigo and pyoderma, cellulitis, abscess, fungal infection, lice and other skin infections [20] (S1 Table). Inter-hospital transfers were combined into a single hospital admission. Readmissions within 14 days of discharge were combined into one episode of infection.

### Statistical analysis

Age specific skin infection admission rates per 1000 child-years and their 95% confidence intervals (CI) were calculated. Child-years at risk for Aboriginal and non-Aboriginal children in various age groups and geographical regions were calculated using dates of birth, dates of death and the end of the study period (31 December 2012). Skin infection cases were defined using the principal and additional diagnoses fields of hospital records, unless otherwise stated. Admissions rates grouped by age groups were presented separately for Aboriginal and non-Aboriginal children, by geographical location and socio-economic status. Aboriginal children were identified using the ‘Getting Our Story Right’ flag (GOSR) [21]. GOSR is a validated flag based on published algorithms to identify Aboriginal status across numerous administrative datasets. This widely used algorithm is used to reduce missing data and to ensure consistent, reliable recording of Aboriginal status [21]. The mother’s postcode at the time of her child’s birth was used to define the geographical location and socio-economic status. We used the Index of Relative Socio-economic Advantage and Disadvantage defined by the Australian Bureau of Statistics to stratify the population into socio-economic quantiles ranging from most-disadvantaged to least-disadvantaged [22]. Median differences in admission age were compared using the non-parametric equality of medians tests, and proportional difference in length of stay was compared using the Mann-Whitney test. Year to year percentage changes in admission rates from 1996 to 2012, were calculated using log-linear modelling with negative-binomial regression. Deviation from uniform distribution of admissions across seasons was analyzed by stratifying admissions by month, and testing for statistical significance using the Chi-square test of seasonality. A P < 0.05 was considered significant. Seasonal differences in principal skin infections admissions were investigated in the Kimberley and Perth metropolitan areas due to their distinct seasonal patterns. Perth metropolitan is part of the Southern hemisphere with seasons being: summer (December–February), autumn (March–May), winter (June–August) and spring (September—November). In the Kimberley, the seasons are bimodal, with a wet (November–April) and dry season (May–October). Data analysis was performed using SPSS (version 23), EpiBasic (version 2) and STATA (version 13.1).

### Ethical approval

This study was part of (i) a larger program of work to assess the pathogen-specific burden of acute lower respiratory infections in children using skin infections as a non-vaccine preventable infection control group in order to understand temporal trends in hospital admissions in light of targeted vaccination programs; and (ii) a PhD project documenting the public health significance of skin infections in remote WA. The study was approved by the Western Australian Department of Health Human Research Ethics Committee, the Western Australian Aboriginal Health Ethics Committee and the University of Western Australia Human Research Ethics Committee.

## Results

### Study population

Our birth cohort consisted of 469,589 children born between 1996 and 2012. Of these, 31,348 (6.7%) were Aboriginal and 240,237 (51.2%) were boys. Singleton births accounted for 97.0% of the cohort and 2538 children (0.5%) were deceased by 2012. There were 15,377 hospital admissions for skin infection in children aged <16 years, accounting for 2.8% of 541,297 hospital admissions between 1996–2012. Aboriginal children had a 15.0 (95% CI 14.5–15.5) times higher admission rate for skin infection (31.7/1000 child-years) than non-Aboriginal children (2.1/1000 child-years). The proportion of children hospitalized for skin infection was significantly higher in Aboriginal children compared to non-Aboriginal children (14.8% vs. 1.5%; odds ratio [OR] 11.3; 95%CI 10.9–11.7). Multiple admissions per child for skin infection were more common in Aboriginal children (OR 3.8; 95% CI: 3.4–4.1). Admissions across all age groups were 1.1 (95% CI 1.1–1.2) times higher in males than females. The median age at admission was younger in Aboriginal children (26.0 months (interquartile range [IQR]: 10.0–64.0 months) vs. 35.0 months (IQR: 14.0–75.0 months)) in non-Aboriginal children. The mean length of stay was longer for Aboriginal children compared to non-Aboriginal children (7.3 days v 4.8 days; P <0.001). Children from most socio-economically disadvantaged areas had higher admission rates compared to children from the least socio-economically disadvantaged areas (IRR 2.3; 95% CI 1.4–3.8 for Aboriginal children and IRR 2.1; 95% CI 1.8–2.3 for non-Aboriginal children) (Table 1).

**Table 1:** Admission rates for skin infection in Western Australian Aboriginal and non-Aboriginal children, by socioeconomic status, 1996–2012.

	Aboriginal			Non-Aboriginal	
IRSAD^a^	Rate/1000	IRR^b^ (95% CI)		Rate/1000	IRR^b^ (95% CI)
91–100%	14.9	Ref		1.5	Ref
76–90%	19.2	1.3 (0.8–2.2)		1.7	1.1 (1.0–1.3)
26–75%	25.4	1.7 (1.0–2.8)		2.3	1.5 (1.4–1.7)
11–25%	23.8	1.6 (1.0–2.6)		2.5	1.7 (1.5–1.9)
0–10%	34.7	2.3 (1.4–3.8)		3.1	2.1 (1.8–2.3)

a) Index of Relative Socioeconomic Advantage and Disadvantage; 90–100%, least disadvantaged; 0–10%, most disadvantaged.

b) Rate of admission/1000 child-years.

CI, Confidence interval.

### Principal diagnosis of skin infection

Admissions with a skin infection associated principal diagnosis accounted for 59.4% of the total skin infection admissions. In Aboriginal children, abscess was the most common principal diagnosis (42.2%) followed by cellulitis (26.0%), scabies (15.8%), impetigo and pyoderma (14.3%), fungal infection (1.1%) and head lice (0.7%). In non-Aboriginal children, cellulitis was the most common principal diagnosis (52.8%), followed by abscess (33.3%), impetigo and pyoderma (12.5%), scabies (0.8%), fungal infection (0.4%) and head lice (0.2%). In children aged <16 years the hospitalization rate of skin infection by principal diagnosis was 17.5/1000 (95% CI 17.0–18.0) in Aboriginal children and 1.4/1000 (95% CI 1.3–1.4) in non-Aboriginal children. 87.6% of admissions for any skin infection as principal diagnosis were emergency admissions. Where skin infections were coded as an additional diagnosis, the principal reason for hospital admission was most commonly due to respiratory and gastrointestinal infections (27.3%).

### Temporal trends

**Fig 2:**
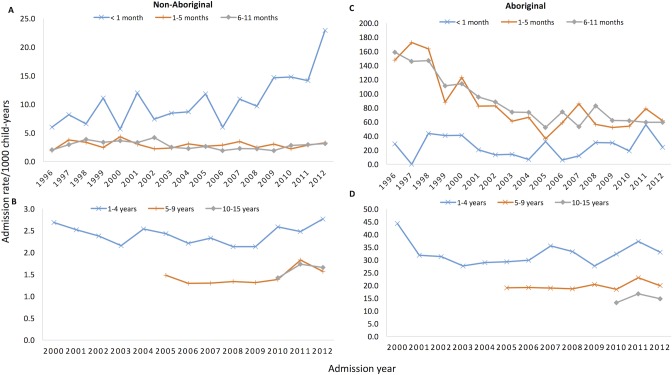
Skin infection hospital admission rates in children in Western Australia, 1996–2012. Shows rates in non-Aboriginal (A & B) and Aboriginal (C & D) children. The calculated admissions included all hospital discharge records with evidence of scabies, impetigo and pyoderma, cellulitis, abscess, fungal infections, head lice and other skin infections. Note the differences in scale. In children aged 1–4 years, the admission rate of skin infection was 34.1/1000 in Aboriginal children and 2.5/1000 in non-Aboriginal children (Table 2). In Aboriginal children of this age group, significant declines were mainly seen in admissions for scabies (4.2%), impetigo and pyoderma (2.7%), and cellulitis (5.0%). Conversely, abscess increased by 2.1% in Aboriginal children (P = 0.03). In children aged 5–9 years and 10–15 years, the highest rates of admissions were for abscess, while the highest disparity in admissions between Aboriginal and non-Aboriginal was due to scabies. There were no significant changes in admission rates during the follow-up period in both these age groups.

Hospital admissions for skin infections were significantly higher in infants aged <1 year throughout the study period, with the rate 22.5 times higher in Aboriginal infants (78.9/1000; 95% CI 75.8–82.1) than non-Aboriginal infants (3.5/1000; 95% CI 3.4–3.7; Table 2). Among Aboriginal infants, hospital admission rates were highest in those aged 6–11 months (83.8/1000; 95% CI 78.8–88.9) whereas for non-Aboriginal infants the rates were highest among those aged <1 month (10.9/1000; 95% CI 9.9–12.1) (Fig 2A & 2C). The rate in Aboriginal infants significantly declined by 6.2%/year over the study period in those aged 1–5 months and by 6.2%/year in those aged 6–11 months (both P <0.001; Fig 2C). The declines were predominately observed in admissions for cellulitis (4.9%/year; P <0.004), and scabies (8.9%/year; both P <0.001). The highest disparity in admission rates between Aboriginal and non-Aboriginal was in scabies admissions among infants (IRR 417.0; 95% CI 308.8–576.7). Cellulitis and abscess accounted for the lowest disparity between Aboriginal and non-Aboriginal.

**Table 2:** Age-specific hospitalisations in Western Australian Aboriginal and non-Aboriginal children discharged with a principal and/or additional diagnosis of skin infections, 1996–2012.

Skin condition*	Age	Aboriginal		Non-Aboriginal		IRR^b^	95% CI
		No.	Rate^a^	No.	Rate^a^		
Cellulitis	<1 year	396	13.1	583	1.4	9.5	(8.4–10.8)
	1–4 years	992	9.8	2045	1.5	6.7	(6.2–7.2)
	5–9 years	513	6.3	978	0.9	7.2	(6.5–8.0)
	10–15 years	220	5.5	492	0.9	6.5	(5.5–7.6)
Abscess	<1 year	285	9.6	378	0.9	10.7	(9.0–12.4)
	1–4 years	1052	10.4	770	0.7	18.9	(17.2–20.7)
	5–9 years	760	9.3	433	0.4	23.9	(21.3–27.0)
	10–15 years	268	6.7	308	0.6	12.3	(10.4–14.6)
Impetigo & Pyoderma	<1 year	707	23.8	398	0.9	24.9	(22.0–28.2)
	1–4 years	887	8.8	556	0.4	22.0	(19.8–24.5)
	5–9 years	231	2.8	193	0.2	16.3	(13.4–19.9)
	10–15 years	74	1.9	71	0.1	14.8	(10.5–20.7)
Scabies	<1 year	1309	43.6	44	0.1	417.0	(308.8–576.7.6)
	1–4 years	735	7.3	43	0.0	235.9	(173.4–328.83)
	5–9 years	160	2.0	12	0.0	181.9	(101.3–359.4)
	10–15 years	50	1.3	<5	0.0	176.8	(64.9–674.3)
Head Lice	<1 year	72	2.4	8	0.0	126.1	(60.7–303.3)
	1–4 years	388	3.9	128	0.1	41.8	(34.8–51.5)
	5–9 years	283	3.5	98	0.1	39.4	(31.2–50.1)
	10–15 years	81	2.0	38	0.1	30.2	(20.3–45.6)
Fungal infections	<1 year	259	8.6	154	0.4	23.6	(19.2–29.0)
	1–4 years	257	2.6	124	0.1	28.6	(23.0–35.7)
	5–9 years	79	1.0	35	0.0	30.8	(20.4–47.2)
	10–15 years	18	0.5	23	0.0	11.1	(5.6–21.4)
All Skin Infections	<1 year	2371	78.9	1493	3.5	22.3	(20.9–23.8)
	1–4 years	3425	34.1	3437	2.5	13.8	(13.1–14.4)
	5–9 years	1625	19.8	1612	1.4	13.8	(12.8–14.7)
	10–15 years	578	14.5	836	1.5	9.8	(8.8–10.9)

a) Rate of admission per 1000 child-years.

b) IRR = Relative rate of Aboriginal to non-Aboriginal admission rate.

CI, confidence interval.

*Note the sum of the individual diagnostic categories doesn’t add up to the total skin infections, as some patients present with multiple skin infections.

### Geographical and seasonal variations in admissions

The highest rates of admissions for all skin infections in Aboriginal children aged <16 years were observed in the remote regions (Pilbara (46.9/1000; 95% CI 44.2–49.7), Kimberley (45.3/1000; 95% CI 43.6–47.0), and Goldfields (42.1/1000; 95% CI 39.1–44.9). This finding was consistent for every type of skin infection. In rural regions, the rate in Aboriginal children aged <16 years was highest in the Midwest-Murchison area (31.1/1000; 95% CI 29.2–33.2) (Table 3). The highest disparities in admissions between Aboriginal and non-Aboriginal children were observed in infants aged <1 years in remote regions (Table 4). Whilst hospitalization rates remained high, there was a declining trend over time across all geographical areas. The overall hospitalization rate in Aboriginal infants aged <1 year declined significantly by 7.5%/year in the metropolitan region, 5.3%/year in rural and 5.3%/year in remote regions (all P <0.001) over the study period.

**Table 3:** Number and rate of hospital admissions for skin infections^a^ in WA-born Aboriginal and non-Aboriginal children aged <16 years, by region, 1996–2012.

	**Aboriginal**			**Non-Aboriginal**		
	**n**	**Rate/1000**^**b**^	**(95% CI)**	**n**	**Rate/1000**^**b**^	**(95% CI)**
**Metropolitan Areas**						
<1 years	507	46.6	(42.7–50.9)	1176	3.6	(3.4–3.8)
1–4 years	779	21.6	(20.1–23.2)	2697	2.5	(2.4–2.6)
5–9 years	408	14.1	(12.8–15.6)	1225	1.4	(1.4–1.5)
10–15 years	170	12.1	(10.4–14.1)	624	1.5	(1.4–1.6)
<16 years	1864	20.8	(19.8–21.7)	5722	2.2	(2.1–2.2)
**Kimberley**						
<1 years	797	113.3	(105.6–121.4)	13	3.3	(1.8–5.6)
1–4 years	1189	49.5	(46.8–52.4)	41	3.1	(2.2–4.2)
5–9 years	587	28.9	(26.6–31.3)	27	2.5	(1.6–3.6)
10–15 years	208	20.8	(18.1–23.8)	9	1.7	(0.8–3.2)
<16 years	2781	45.3	(43.6–47.0)	90	2.7	(2.1–3.3)
**Pilbara**						
<1 years	373	129.8	(116.9–143.6)	39	4.2	(3.0–5.7)
1–4 years	508	53.7	(49.1–58.6)	88	2.8	(2.3–3.5)
5–9 years	184	23.2	(20.0–26.8)	35	1.3	(0.9–1.9)
10–15 years	71	17.9	(14.0–22.6)	17	1.2	(0.7–1.9)
<16 years 1136 46.9 (44.2–49.7) 179 2.2						(1.9–2.6)
**Midwest-Murchison**						
<1 years	273	73.6	(65.1–82.7)	44	3.5	(2.6–4.7)
1–4 years	422	33.7	(30.6–37.1)	107	2.5	(2.1–3.0)
5–9 years	209	21.1	(18.4–24.2)	63	1.7	(1.3–2.2)
10–15 years	59	12.3	(9.4–15.9)	41	2.0	(1.5–2.8)
<16 years 963 31.1 (29.2–33.2) 255 2.3						(2.0–2.6)
**South West**						
<1 years	25	21.9	(14.6–32.3)	85	2.9	(2.4–3.6)
1–4 years	42	11.1	(8.0–15.0)	204	2.1	(1.9–2.5)
5–9 years	22	7.1	(4.4–10.7)	97	1.3	(1.0–1.5)
10–15 years	11	7.1	(3.5–12.7)	45	1.2	(0.9–1.6)
<16 years	100	10.4	(8.5–12.7)	431	1.8	(1.6–2.0)
**Goldfields**						
<1 years	299	131.8	(117.3–147.6)	54	3.9	(2.9–5.1)
1–4 years	344	44.5	(39.9–49.5)	134	2.8	(2.4–3.3)
5–9 years	139	21.8	(18.3–25.7)	54	1.3	(1.0–1.7)
10–15 years	38	12.2	(8.6–16.8)	32	1.4	(1.0–2.0)
<16 years 820 42.1 (39.2–45.1) 270 2.8						(1.9–2.5)
**Great Southern**						
<1 years	37	41.9	(29.5–57.7)	30	2.6	(1.8–3.8)
	Aboriginal			Non-Aboriginal		
	n	Rate/1000^b^	(95% CI)	n	Rate/1000^b^	(95% CI)
1–4 years	45	15.1	(11.0–20.2)	62	1.6	(1.3–2.1)
5–9 years	32	12.9	(8.8–18.2)	41	1.3	(0.9–1.7)
10–15 years	13	11.0	(5.9–18.8)	19	1.1	(0.6–1.6)
<16 years	127	16.9	(14.1–20.1)	152	1.5	(1.3–1.8)
**Wheatbelt**						
<1 years	53	36.9	(27.7–48.3)	51	3.3	(2.4–4.3)
1–4 years	76	15.9	(12.5–19.9)	101	6.1	(1.4–2.2)
5–9 years	37	9.7	(6.8–13.4)	65	1.2	(1.1–1.8)
10–15 years	7	3.9	(1.6–8.0)	49	1.0	(1.4–2.5)
<16 years	173	14.6	(12.5–17.0)	266	1.9	(1.6–2.1)

a) Any mention of skin infection in the principal and additional diagnoses fields.

b) Rate of hospitalisations per 1000 child-years, WA, Western Australia

**Table 4:** Hospital admissions for skin infections* in Aboriginal and non-Aboriginal children born in WA between 1996–2012, by age and WA region of residence.

Age Group	Non-Aboriginal (438, 241)			Aboriginal (31,348)				
	No.	Rate^a^	Regional IRR^b^ (95% CI)	No.	Rate^a^	Regional IRR^b^ (95% CI)	IRR^c^	(95% CI)
**<1 month**								
Metropolitan	316	11.4	1	17	18.6	1	1.6	(1.0–2.7)
Rural	51	8.8	0.8 (0.6–1.0)	8	13.3	0.7 (0.3–1.7)	1.5	(0.7–3.2)
Remote	24	10.4	0.9 (0.6–1.4)	38	37.3	2.0 (1.1–3.6)	3.6	(2.1–6.0)
**1–5 months**								
Metropolitan	404	2.9	1	247	54.1	1	18.5	(15.8–21.6)
Rural	79	2.7	0.9 (0.7–1.2)	177	58.8	1.1 (0.9–1.3)	21.5	(16.5–28.1)
Remote	36	3.1	1.1 (0.8–1.5)	641	125.8	2.3 (2.0–2.7)	40.2	(28.7–56.3)
**6–11 months**								
Metropolitan	456	2.9	1	243	46.2	1	16.1	(13.8–18.8)
Rural	80	2.4	0.8 (0.7–1.1)	203	58.3	1.3 (1.1–1.5)	24.5	(18.9–31.7)
Remote	46	3.4	1.2 (0.9–1.6)	790	133.6	2.9 (2.5–3.3)	38.8	(28.8–52.2)
**1–4 years**								
Metropolitan	2697	2.5	1	779	22.1	1	8.6	(7.9–9.3)
Rural	474	2.1	0.8 (0.7–0.9)	585	24.7	1.1 (1.0–1.3)	12.0	(10.6–13.6)
Remote	263	2.9	1.1 (1.0–1.3)	2041	50.5	2.3 (2.1–2.5)	17.6	(15.5–20.1)
**5–9 years**								
Metropolitan	1225	1.5	1	408	14.4	1	9.9	(8.8–11.1)
Rural	266	1.4	0.9 (0.8–1.1)	300	15.8	1.1 (1.0–1.3)	11.4	(9.7–13.5)
Remote	116	1.5	1.02 (0.9–1.2)	910	26.9	1.9 (1.7–2.1)	18.1	(14.9–22.0)
**10–15 years**								
Metropolitan	624	1.5	1	170	12.4	1	8.4	(7.3–10.2)
Rural	154	1.5	1.0 (0.9–1.2)	90	9.8	0.8 (0.6–1.0)	6.4	(5.0–8.4)
Remote	58	1.4	0.9 (0.7–1.2)	317	19.1	1.5 (1.3–1.9)	10.5	(10.5–18.3)

a) Rate of admission/1000 child-years.

b) Regional IRR = relative rate of rural/remote to metropolitan admission rate.

c) IRR = relative rate of Aboriginal to non-Aboriginal admission rate.

* Any mention of skin infection in the principal and additional diagnoses fields.

CI, Confidence interval.

WA, Western Australia.

44 records with missing remoteness information were excluded.

Excess hospitalizations in the number of principal hospital admissions for skin infection were observed among Aboriginal children during the wet season in the Kimberley region (P <0.001) and during summer in the Perth metropolitan areas (P = 0.04). These increases were predominately due to scabies, abscess and cellulitis in children aged 1–4 years.

## Discussion

This is the first in-depth analysis of linked hospitalization data to describe the burden and epidemiology of skin infections for a birth cohort of an entire state of Australia. Our study is based on 17 years of hospitalization data, incorporating > 6 million child years at risk. We report three key findings: (i) Aboriginal children were 15 times (22.5 times for infants) more likely to be hospitalized with a skin infection than non-Aboriginal children; (ii) the highest admission rates for skin infections were in infants aged <1 year (8 out of every 100 Aboriginal children were hospitalized with a skin infection in the first year of life); (iii) skin infections are not just a primary care issue, but also represent a substantial burden on the hospital system (3 out of every 100 child hospital admissions). Improving primary care of skin infections is likely to reduce this hospitalization burden and improve health and wellbeing outcomes for Aboriginal children.

We confirmed a very high burden of hospital admissions due to skin infections in infants aged <1 year in both Aboriginal and non-Aboriginal children. Admission rates for this age group were 2.3 and 1.4 times higher for Aboriginal and non-Aboriginal children respectively when compared to the age group with the second highest skin infection hospitalization rate (1 to 4 year olds). Although previously published hospital admission data have documented a similar trend in certain settings and for specific skin conditions [9,10,23], to our knowledge this is the first birth cohort study to highlight the steep burden of skin infections leading to hospitalization in infants. Other studies of skin infection related hospitalizations in children from Turkey [24], New Zealand [25–28] and the USA [29] used wider ranges for their youngest age groups. Our data are consistent with previous observations from remote Australian Aboriginal communities that showed high rates of primary care presentations for skin infections in infants, particularly in the first few months of life [30–32]. We confirm this with high hospitalization rates for skin infections throughout the first year of life, particularly beyond the neonatal period. It is possible that this high incidence of skin infection associated hospitalizations is driven by scabies infestations in neonates, making them susceptible to secondary bacterial skin infections in infancy [33].

We show that hospitalization rates in Aboriginal infants (aged 1 to 12 months) have been steadily declining, as the gap with the lower rates observed in Aboriginal neonates in the first month of life -although still considerable- has narrowed. This decline was also observed in the 1-to-4 year age group of Aboriginal children and may be consistent with improvements in primary health care access and delivery, a different threshold for hospitalization, or overall improving trends in the burden of infectious diseases in infancy. We now plan to investigate the temporal trends in hospitalization rates for other infections, namely acute lower respiratory infections, which can be compared with these trends presented here. Interestingly, and counter to our observations in Aboriginal children, skin-related hospital admissions for non-Aboriginal children peaked during the first 30 days of life. Furthermore, our data shows an upward trend in skin infections in this particular group of neonates. These observations are possibly consistent with increasing admissions for omphalitis, staphylococcal scalded skin and periungual cellulitis in the early post-natal period in non-Aboriginal children [34,35].

Aboriginal children are more likely to be admitted to hospital for skin infection, stay longer and have more episodes of abscess. This is consistent with community prevalence studies that confirm a high, sustained burden of skin infections in Australian Aboriginal children [2,5]. Hospital admission data only captures a segment of health service utilization associated with skin infections. A study set in a disadvantaged region of New Zealand estimated that for every one skin infection related hospitalization there were 14 primary care cases [36], further illustrating that skin infections are predominantly a primary care issue. In Australia, other datasets confirm a high burden of skin infections at the primary care level for Aboriginal Australians (6.6 out of every 100 general practitioner consultations, compared to 2.1 for non-Aboriginal Australians) [37]. Furthermore, as confirmed in our analysis, the burden of skin infections in children is highest in remote Aboriginal communities [2,8,30–32]. This is illustrative of the overall burden of infectious diseases in remote Aboriginal communities, which has been associated with a wide range of health service, sociocultural and environmental factors, including high primary care staff turnover rates, socioeconomic disadvantage and poor housing conditions [5].

We observed seasonal trends in skin infection hospitalization rates among Aboriginal children living in the Kimberley (tropical and sub-tropical climate) and the Perth metropolitan area (temperate climate). Seasonality trends in consultation and hospitalization rates for bacterial skin infections have previously been observed in temperate and tropical areas, with peak incidences often occurring in summer and autumn [25,38–40]. Factors related to pathogen survival, vector abundance, host behavior and immune function might underpin such seasonal variation [41]. Our data also show that skin infection associated hospitalization rates for Aboriginal children living in the Kimberley are at their highest during the tropical, high humidity months of the year. It is possible that these conditions may promote microbial growth on the skin [42–45], increase the risk of infection-prone insect bites [46–50] and contribute to the survival and transmission of scabies mites [51–54].

This vast dataset constitutes 17 years of hospitalization data for almost 500,000 children. We have found important socio-demographic trends, with infants and Aboriginal children at a much higher risk of developing skin infections requiring tertiary care. The limitation of analyzing a hospitalization dataset is that it only captures the severe end of the disease burden, since skin infections remain a primary care issue first and foremost [2,36]. Despite the high reported burden, lack of clinical documentation about skin infections in the hospital record may underestimate the true burden of skin infections [55]. This suggests that skin infections are underdiagnosed in hospital settings, presumably due to under-recognition or normalization associated with the high ongoing burden of skin infections.

Our data demonstrate that in Australia, Aboriginal children living in rural and remote areas are at a disproportionately high risk of being hospitalized for skin infections. These findings are in line with previous studies that have documented an extremely high prevalence of skin disease in children living in these settings [2]. The impact of skin infections in remote communities extends well beyond the need for acute care, as its ubiquity affects childhood development [56], poses a risk for developing other acute and chronic health conditions [45] and incurs a significant cost to the public health system when hospitalization is required [10]. Reducing the need for hospitalization as the end-point in care through improved community-level prevention of skin infections and the provision of effective primary care are crucial.

## Supporting information

S1 Table ICD-9-CM and ICD-10-AM diagnosis codes used to identify hospital admission for skin infections. (DOCX)

## References

[1] Hay RJ, Johns NE, Williams HC, Bolliger IW, Dellavalle RP, Margolis DJ, et al The global burden of skin disease in 2010: an analysis of the prevalence and impact of skin conditions. J Invest Dermatol. Elsevier; 2014;134: 1527–34. doi: 10.1038/jid.2013.446 24166134 10.1038/jid.2013.446

[2] Bowen AC, Mahé A, Hay RJ, Andrews RM, Steer AC, Tong SYC, et al The global epidemiology of impetigo: A systematic review of the population prevalence of impetigo and pyoderma. PLoS One. 2015;10: 1–15. doi: 10.1371/journal.pone.0136789 26317533 10.1371/journal.pone.0136789 PMC4552802

[3] Carapetis JR, Steer AC, Mulholland EK, Weber M. The global burden of group A streptococcal diseases. Lancet Infect Dis. 2005;5: 685–694. doi: 10.1016/S1473-3099(05)70267-X 16253886 10.1016/S1473-3099(05)70267-X

[4] Romani L, Steer AC, Whitfeld MJ, Kaldor JM. Prevalence of scabies and impetigo worldwide: a systematic review. Lancet Infect Dis. Elsevier Ltd; 2015;15: 960–967. doi: 10.1016/S1473-3099(15)00132-2 26088526 10.1016/S1473-3099(15)00132-2

[5] Quinn EK, Massey PD, Speare R. Communicable diseases in rural and remote Australia: the need for improved understanding and action. Rural Remote Heal. 2015;15: 1–19. doi: 10.1613/jair.301 26391139

[6] Currie BJ, Carapetis JR. Skin infections and infestations in Aboriginal communities in northern Australia. Australas J Dermatol. 2000; 139–145. 10954983 10.1046/j.1440-0960.2000.00417.x

[7] Lehmann D, Tennant MT, Silva DT, McAullay D, Lannigan F, Coates H, et al Benefits of swimming pools in two remote Aboriginal communities in Western Australia: intervention study. BMJ. 2003;327: 415–9. doi: 10.1136/bmj.327.7412.415 12933727 10.1136/bmj.327.7412.415 PMC181254

[8] Silva DT, Lehmann D, Tennant MT, Jacoby P, Wright H, Stanley FJ. Effect of swimming pools on antibiotic use and clinic attendance for infections in two Aboriginal communities in Western Australia. Med J Aust. 2008;188: 594–8. Available: http://www.ncbi.nlm.nih.gov/pubmed/18484935 18484935 10.5694/j.1326-5377.2008.tb01800.x

[9] Vaska VL, Nimmo GR, Jones M, Grimwood K, Paterson DL. Increases in Australian cutaneous abscess hospitalisations: 1999–2008. Eur J Clin Microbiol Infect Dis. 2012;31: 93–6. doi: 10.1007/s10096-011-1281-3 21553298 10.1007/s10096-011-1281-3

[10] Whitehall J, Kuzulugil D, Sheldrick K, Wood A. Burden of paediatric pyoderma and scabies in North West Queensland. J Paediatr Child Health. 2013;49: 141–3. doi: 10.1111/jpc.12095 23347222 10.1111/jpc.12095

[11] Carville KS, Lehmann D, Hall G, Moore H, Richmond P, de Klerk N, et al Infection is the major component of the disease burden in aboriginal and non-aboriginal Australian children: a population-based study. Pediatr Infect Dis J. 2007;26: 210–6. Available: http://www.ncbi.nlm.nih.gov/pubmed/17484216 17484216 10.1097/01.inf.0000254148.09831.7f

[12] Australian Bureau of Statistics. 3238.0.55.001—Estimates of Aboriginal and Torres Strait Islander Australians, June 2011 [Internet]. 2013 [cited 12 Nov 2016]. Available: http://www.abs.gov.au/ausstats/abs@.nsf/mf/3238.0.55.001

[13] Australian Bureau of Statistics. 3235.0—Population by Age and Sex, Regions of Australia, 2014 [Internet]. 2014 [cited 8 Aug 2016]. Available: http://www.abs.gov.au/AUSSTATS/abs@.nsf/DetailsPage/3235.02014?OpenDocument

[14] Australian Institute of Health and Welfare. Rural, regional and remote health: a guide to remoteness classifications [Internet]. Canberra; 2004. Available: http://www.aihw.gov.au/WorkArea/DownloadAsset.aspx?id=6442459567

[15] Commissioner for Children and Young People. The State of Western Australia’s Children and Young People—Edition Two [Internet]. Perth; 2014. Available: http://www.rdagreatsouthern.com.au/pdf/TheStateofWesternAustraliasChildrenandYoungPeople-EditionTwo-14July2014.pdf

[16] Hogan AB, Anderssen RS, Davis S, Moore HC, Lim FJ, Fathima P, et al Time series analysis of RSV and bronchiolitis seasonality in temperate and tropical Western Australia. Epidemics. Elsevier B.V.; 2016;16: 49–55. doi: 10.1016/j.epidem.2016.05.001 27294794 10.1016/j.epidem.2016.05.001

[17] Holman CDJ, Bass AJ, Rosman DL, Smith MB, Semmens JB, Glasson EJ, et al A decade of data linkage in Western Australia: strategic design, applications and benefits of the WA data linkage system. Aust Health Rev. 2008;32: 766–77. Available: http://www.ncbi.nlm.nih.gov/pubmed/18980573 18980573 10.1071/ah080766

[18] Holman CD, Bass AJ, Rouse IL, Hobbs MS. Population-based linkage of health records in Western Australia: development of a health services research linked database. Aust N Z J Public Health. 1999;23: 453–9. Available: http://www.ncbi.nlm.nih.gov/pubmed/10575763 10575763 10.1111/j.1467-842x.1999.tb01297.x

[19] Kelman CW, Bass AJ, Holman CDJ. Research use of linked health data—a best practice protocol. Aust N Z J Public Health. 2002;26: 251–5. Available: http://www.ncbi.nlm.nih.gov/pubmed/12141621 12141621 10.1111/j.1467-842x.2002.tb00682.x

[20] Burgner DP, Cooper MN, Moore HC, Stanley FJ, Thompson PL, de Klerk NH, et al Childhood hospitalisation with infection and cardiovascular disease in early-mid adulthood: a longitudinal population-based study. PLoS One. 2015;10: e0125342 doi: 10.1371/journal.pone.0125342 25938548 10.1371/journal.pone.0125342 PMC4418819

[21] Christensen D, Davis G, Draper G, Mitrou F, McKeown S, Lawrence D, et al Evidence for the use of an algorithm in resolving inconsistent and missing Indigenous status in administrative data collections. Aust J Soc Issues,. Australian Social Policy Association; 2014;49: 423.

[22] Australian Bureau of Statistics. Census of Population and Housing: Socio-Economic Indexes for Areas (SEIFA), Australia. Canberra; 2013.

[23] Seale AC, Davies MR, Anampiu K, Morpeth SC, Nyongesa S, Mwarumba S, et al Invasive Group A Streptococcus Infection among Children, Rural Kenya. Emerg Infect Dis. Centers for Disease Control and Prevention; 2016;22: 224–32. doi: 10.3201/eid2202.151358 26811918 10.3201/eid2202.151358 PMC4734542

[24] Tamer E, Ilhan MN, Polat M, Lenk N, Alli N. Prevalence of skin diseases among pediatric patients in Turkey. J Dermatol. 2008;35: 413–8. doi: 10.1111/j.1346-8138.2008.00495.x 18705828 10.1111/j.1346-8138.2008.00495.x

[25] O’Sullivan CE, Baker MG, Zhang J. Increasing hospitalizations for serious skin infections in New Zealand children, 1990–2007. Epidemiol Infect. 2011;139: 1794–1804. doi: 10.1017/S0950268810002761 21156094 10.1017/S0950268810002761

[26] O’Sullivan C, Baker MG. Serious skin infections in children: a review of admissions to Gisborne Hospital (2006–2007). N Z Med J. 2012;125: 55–69. Available: http://www.ncbi.nlm.nih.gov/pubmed/22426611 22426611

[27] O’Sullivan C, Baker MG, Zhang J, Davies A, Cramp G. The epidemiology of serious skin infections in New Zealand children: comparing the Tairawhiti region with national trends. N Z Med J. 2012;125: 40–54. Available: http://www.ncbi.nlm.nih.gov/pubmed/22426610 22426610

[28] Lim A, Rumball-Smith J, Jones R, Kawachi I. The rise and fall of hospitalizations for skin infections in New Zealand, 2004–2014: trends by ethnicity and socioeconomic deprivation. Epidemiol Infect. 2017;145: 678–684. doi: 10.1017/S0950268816002685 27903309 10.1017/S0950268816002685 PMC9507719

[29] Miller LG, Eisenberg DF, Liu H, Chang C-L, Wang Y, Luthra R, et al Incidence of skin and soft tissue infections in ambulatory and inpatient settings, 2005–2010. BMC Infect Dis. BioMed Central; 2015;15: 362 doi: 10.1186/s12879-015-1071-0 26293161 10.1186/s12879-015-1071-0 PMC4546168

[30] Clucas DB, Carville KS, Connors C, Currie BJ, Carapetis JR, Andrews RM. Disease burden and health-care clinic attendances for young children in remote aboriginal communities of northern Australia. Bull World Health Organ. 2008;86: 275–81. Available: http://www.pubmedcentral.nih.gov/articlerender.fcgi?artid=2647416&tool=pmcentrez&rendertype=abstract doi: 10.2471/BLT.07.043034 18438516 10.2471/BLT.07.043034 PMC2647416

[31] McMeniman E, Holden L, Kearns T, Clucas DB, Carapetis JR, Currie BJ, et al Skin disease in the first two years of life in Aboriginal children in East Arnhem Land. Australas J Dermatol. 2011;52: 270–3. Available: http://www.ncbi.nlm.nih.gov/pubmed/22070701 doi: 10.1111/j.1440-0960.2011.00806.x 22070701 10.1111/j.1440-0960.2011.00806.x

[32] Kearns T, Clucas D, Connors C, Currie BJ, Carapetis JR, Andrews RM. Clinic attendances during the first 12 months of life for Aboriginal children in five remote communities of northern Australia. PLoS One. 2013;8: e58231 doi: 10.1371/journal.pone.0058231 23469270 10.1371/journal.pone.0058231 PMC3585931

[33] Tong SYC, Steer AC, Jenney AW, Carapetis JR. Community-associated methicillin-resistant Staphylococcus aureus skin infections in the tropics. Dermatol Clin. 2011;29: 21–32. Available: http://www.ncbi.nlm.nih.gov/pubmed/21095524 doi: 10.1016/j.det.2010.09.005 21095524 10.1016/j.det.2010.09.005

[34] Stewart D, Benitz W, COMMITTEE ON FETUS AND NEWBORN. Umbilical Cord Care in the Newborn Infant. Pediatrics. 2016;138 doi: 10.1542/peds.2016-2149 27573092 10.1542/peds.2016-2149

[35] Mishra AK, Yadav P, Mishra A. A Systemic Review on Staphylococcal Scalded Skin Syndrome (SSSS): A Rare and Critical Disease of Neonates. Open Microbiol J. 2016;10: 150–9. doi: 10.2174/1874285801610010150 27651848 10.2174/1874285801610010150 PMC5012080

[36] O’Sullivan C, Baker MG. Skin infections in children in a New Zealand primary care setting: exploring beneath the tip of the iceberg. N Z Med J. 2012;125: 70–9. Available: http://www.ncbi.nlm.nih.gov/pubmed/22426612 22426612

[37] Fahridin S, Britt H. Aboriginal and Torres Strait Islander patients In: Britt H, Miller G, editors. General practice in Australia, health priorities and policies 1998–2008 General practice series no24 Cat no GEP 24. Canberra: Australian Institute of Heath and Welfare; 2009 pp. 87–104. Available: http://www.aihw.gov.au/publications/index.cfm/title/10721%5Cnhttp://www.aihw.gov.au/publications/gep/gep-24-10721/gep-24-10721-c06.pdf

[38] Mermel LA, Machan JT, Parenteau S. Seasonality of MRSA Infections. DeLeo F, editor. PLoS One. 2011;6: e17925 doi: 10.1371/journal.pone.0017925 21468350 10.1371/journal.pone.0017925 PMC3065770

[39] Loffeld A, Davies P, Lewis A, Moss C. Seasonal occurrence of impetigo: a retrospective 8-year review (1996–2003). Clin Exp Dermatol. 2005;30: 512–4. doi: 10.1111/j.1365-2230.2005.01847.x 16045681 10.1111/j.1365-2230.2005.01847.x

[40] Kristensen JK. Scabies and Pyoderma in Lilongwe, Malawi. Prevalence and seasonal fluctuation. Int J Dermatol. 1991;30: 699–702. Available: http://www.ncbi.nlm.nih.gov/pubmed/1955222 1955222 10.1111/j.1365-4362.1991.tb02612.x

[41] Grassly NC, Fraser C. Seasonal infectious disease epidemiology. Proc R Soc B Biol Sci. The Royal Society; 2006;273: 2541–2550. doi: 10.1098/rspb.2006.3604 16959647 10.1098/rspb.2006.3604 PMC1634916

[42] Singh G. Heat, Humidity and Pyodermas. Dermatology. Karger Publishers; 1973;147: 342–347. doi: 10.1159/000251891 10.1159/000251891 4788478

[43] Taplin D, Zaias N, Rebell G. Environmental influences on the microbiology of the skin. Arch Environ Health. 1965;11: 546–50. Available: http://www.ncbi.nlm.nih.gov/pubmed/5319964 5319964 10.1080/00039896.1965.10664255

[44] Cohen PR. Community-acquired methicillin-resistant Staphylococcus aureus skin infections: a review of epidemiology, clinical features, management, and prevention. Int J Dermatol. 2007;46: 1–11. doi: 10.1111/j.1365-4632.2007.03215.x 17214713 10.1111/j.1365-4632.2007.03215.x

[45] Yeoh DK, Bowen AC, Carapetis JR. Impetigo and scabies–Disease burden and modern treatment strategies. J Infect. Elsevier Ltd; 2016;72: S61–S67. doi: 10.1016/j.jinf.2016.04.024 27180311 10.1016/j.jinf.2016.04.024

[46] Elliot AJ, Cross KW, Smith GE, Burgess IF, Fleming DM. The association between impetigo, insect bites and air temperature: A retrospective 5-year study (1999–2003) using morbidity data collected from a sentinel general practice network database. Fam Pract. 2006;23: 490–496. doi: 10.1093/fampra/cml042 16873392 10.1093/fampra/cml042

[47] Taplin D, Lansdell L, Allen A, Rodriguez R, Cortes A. Prevalence of streptococcal pyoderma in relation to climate and hygiene. Lancet. Elsevier; 1973;301: 501–503. doi: 10.1016/S0140-6736(73)90324-3 10.1016/s0140-6736(73)90324-3 4119945

[48] Hartman-Adams H, Banvard C, Juckett G. Impetigo: diagnosis and treatment. Am Fam Physician. 2014;90: 229–35. Available: http://www.ncbi.nlm.nih.gov/pubmed/25250996 25250996

[49] Meyer Steiger DB, Ritchie SA, Laurance SGW. Mosquito communities and disease risk influenced by land use change and seasonality in the Australian tropics. Parasit Vectors. BioMed Central; 2016;9: 387 doi: 10.1186/s13071-016-1675-2 27388293 10.1186/s13071-016-1675-2 PMC4936001

[50] Franklin DC, Whelan PI. Tropical mosquito assemblages demonstrate “textbook” annual cycles. PLoS One. Public Library of Science; 2009;4: e8296 doi: 10.1371/journal.pone.0008296 20011531 10.1371/journal.pone.0008296 PMC2788620

[51] Davis DP, Moon RD. Survival of Sarcoptes scabiei (De Geer) stored in three media at three temperatures. J Parasitol. 1987;73: 661–2. Available: http://www.ncbi.nlm.nih.gov/pubmed/3110396 3110396

[52] Arlian LG. Biology, host relations, and epidemiology of Sarcoptes scabiei. Annu Rev Entomol. 1989;34: 139–61. doi: 10.1146/annurev.en.34.010189.001035 2494934 10.1146/annurev.en.34.010189.001035

[53] Hay RJ, Steer AC, Engelman D, Walton S. Scabies in the developing world—its prevalence, complications, and management. Clin Microbiol Infect. 2012;18: 313–23. doi: 10.1111/j.1469-0691.2012.03798.x 22429456 10.1111/j.1469-0691.2012.03798.x

[54] Hengge UR, Currie BJ, Jäger G, Lupi O, Schwartz RA. Scabies: a ubiquitous neglected skin disease. Lancet Infect Dis. 2006;6: 769–79. Available: http://www.ncbi.nlm.nih.gov/pubmed/17123897 doi: 10.1016/S1473-3099(06)70654-5 17123897 10.1016/S1473-3099(06)70654-5

[55] Yeoh DK, Anderson A, Cleland G, Banks A, Bowen A. Skin care Assessment in Broome and Port Hedland (SCAB HEAL) project. International Congress for Tropical Medicine and Malaria. Brisbane, Australia; 2016.

[56] Kariuki M, Raudino A, Green MJ, Laurens KR, Dean K, Brinkman SA, et al Hospital admission for infection during early childhood influences developmental vulnerabilities at age 5 years. J Paediatr Child Health. 2016; doi: 10.1111/jpc.13239 27439883 10.1111/jpc.13239

