# Supplementary material for: Hospital admissions for skin infections among Western Australian children and adolescents from 1996 to 2012
Source: PLoS One. 2017 Nov 30;12(11):e0188803. doi: 10.1371/journal.pone.0188803 (PMC5708667; doi:10.1371/journal.pone.0188803)
Supplement: S1 Table — (DOCX) [file pone.0188803.s001.docx]

| **S1 Table.** ICD-9-CM And ICD-10-AM Diagnosis Codes Used to Identify Hospital Admission for Skin Infections | |
| --- | --- |
| **ICD Code** | **Classification** |
| **Scabies** | |
| 133.0 | Scabies (Icd9) |
| B86 | Scabies (ICD10) |
| 133.8 | Acariasis Nec (Icd9) |
| 133.9 | Acariasis Nos (Icd9) |
| B88.0 | Other Acariasis (Icd10) |
|  |  |
| **Impetigo & Pyoderma** | |
| 684 | Impetigo (Icd9) |
| 686.0 | Pyoderma (End 1997) (Icd9) |
| 686.00 | Pyoderma Nos |
| 686.09 | Pyoderma Nec |
| 686.8 | Local Skin Infection Nec |
| 686.9 | Local Skin Infection Nos (Icd9) |
| L01.0 | Impetigo [Any Organism] [Any Site] (Icd10) |
| L08.0 | Pyoderma (ICD10) |
| L01.1 | Impetiginization Of Other Dermatoses |
| L08.8 | Other Specified Local Infections Of Skin And Subcutaneous Tissue |
| L08.9 | Local Infection Of Skin And Subcutaneous Tissue, Unspecified |
| L30.3 | Infective Dermatitis |
| L30.4 | Erythema Intertrigo |
| L00 | Staphylococcal Scalded Skin Syndrome |
|  |  |
| **Fungal** | |
| 110.0 | Dermatophyt Scalp/Beard |
| 110.1 | Dermatophytosis Of Nail |
| 110.2 | Dermatophytosis Of Hand |
| 110.3 | Dermatophytosis Of Groin |
| 110.4 | Dermatophytosis Of Foot |
| 110.5 | Dermatophytosis Of Body |
| 110.6 | Deep Dermatophytosis |
| 110.8 | Dermatophytosis Site Nec |
| 110.9 | Dermatophytosis Site Nos (Icd9) |
| 111.1 | Tinea Nigra |
| 111.2 | Tinea Blanca |
| 111.3 | Black Piedra |
| 111.8 | Dermatomycoses Nec |
| 111.9 | Dermatomycoses Nos |
| 112.3 | Cutaneous Candidiasis (Icd9) |
| B35.0 | Tinea Barbae And Tinea Capitis |
| B35.1 | Tinea Unguium |
| **ICD Code** | **Classification** |
| B35.2 | Tinea Manuum |
| B35.3 | Tinea Pedis |
| B35.4 | Tinea Corporis |
| B35.5 | Tinea Imbricate |
| B35.6 | Tinea Cruris |
| B35.8 | Other Dermatophytoses |
| B35.9 | Dermatophytosis, Unspecified (ICD10) |
| B36.1 | Tinea Nigra |
| B36.2 | White Piedra |
| B36.3 | Black Piedra |
| B36.8 | Other Specified Superficial Mycoses |
| B37.2 | Candidiasis Of Skin And Nail (ICD10) |
| 111.0 | PITYRIASIS VERSICOLOR |
| B36.0 | Pityriasis Versicolour |
| L60.3 | Nail Dystrophy |
|  |  |
| **Abscess** | |
| 680.0 | Carbuncle Of Face (Icd9) |
| 680.1 | Carbuncle Of Neck (Icd9) |
| 680.2 | Carbuncle Of Trunk (Icd9) |
| 680.3 | Carbuncle Of Arm (Icd9) |
| 680.4 | Carbuncle Of Hand |
| 680.5 | Carbuncle Of Buttock (Icd9) |
| 680.6 | Carbuncle Of Leg (Icd9) |
| 680.7 | Carbuncle Of Furuncle Of Foot |
| 680.8 | Carbuncle- Site Nec (Icd9) |
| 680.9 | Carbuncle Nos (Icd9) |
| 685.1 | Pilonidal Cyst W Abscess |
| 685.1 | Pilonidal Cyst W/O Absc (Icd9) |
| 681.01 | Felon |
| 681.02 | Onychia Of Finger |
| 681.11 | Onychia Of Toe |
| L02.0 | Cutaneous Abscess, Furuncle And Carbuncle Of Face (ICD10) |
| L02.1 | Cutaneous Abscess, Furuncle And Carbuncle Of Neck (ICD10) |
| L02.2 | Cutaneous Abscess, Furuncle And Carbuncle Of Trunk (ICD10) |
| L02.3 | Cutaneous Abscess, Furuncle And Carbuncle Of Buttock (ICD10) |
| L02.4 | Cutaneous Abscess, Furuncle And Carbuncle Of Limb (ICD10) |
| L02.8 | Cutaneous Abscess, Furuncle And Carbuncle Of Other Sites (ICD10) |
| L02.9 | Cutaneous Abscess, Furuncle And Carbuncle, Unspecified (ICD10) |
| L05.0 | Pilonidal Cyst With Abscess |
| L05.9 | Pilonidal Cyst Without Abscess (ICD10) |
| H60.0 | Abscess of external ear |
| H60.00 | Abscess of external ear |
| H60.01 | Abscess of external ear |
| H60.02 | Abscess of external ear |
| **ICD Code** | **Classification** |
| H60.03 | Abscess of external ear |
|  |  |
| **Cellulitis** | |
| 681.00 | Cellulitis- Finger Nos (Icd9) |
| 681.10 | Cellulitis- Toe Nos (Icd9) |
| 681.9 | Cellulitis Of Digit Nos |
| 682.0 | Cellulitis Of Face (Icd9) |
| 682.1 | Cellulitis Of Neck (Icd9) |
| 682.2 | Cellulitis Of Trunk (Icd9) |
| 682.3 | Cellulitis Of Arm (Icd9) |
| 682.4 | Cellulitis Of Hand (Icd9) |
| 682.5 | Cellulitis Of Buttock (Icd9) |
| 682.6 | Cellulitis Of Leg (Icd9) |
| 682.7 | Cellulitis Of Foot (Icd9) |
| 682.8 | Cellulitis- Site Nec (Icd9) |
| 682.9 | Cellulitis Nos (Icd9) |
| L03.01 | Cellulitis Of Finger (ICD10) |
| L03.02 | Cellulitis Of Toe (ICD10) |
| L03.10 | Cellulitis Of Upper Limb (ICD10) |
| L03.11 | Cellulitis Of Lower Limb (ICD10) |
| L03.2 | Cellulitis Of Face (ICD10) |
| L03.3 | Cellulitis Of Trunk (ICD10) |
| L03.8 | Cellulitis Of Other Sites (ICD10) |
| L03.9 | Cellulitis, Unspecified (ICD10) |
| 035 | Erysipelas (Icd9) |
| A46 | Erysipelas (ICD10) |
| 380.1 | Infec Otitis Externa Nos (Icd9) |
| 380.11 | Acute Infection Of Pinna (Icd9) |
| H60.1 | Cellulitis of external ear |
| H60.1 | Cellulitis of external ear |
| H60.10 | Cellulitis of external ear |
| H60.11 | Cellulitis of external ear |
| H60.12 | Cellulitis of external ear |
| H60.13 | Cellulitis of external ear |
|  |  |
| **Lice** | |
| 132.0 | Pediculus Capitis (Icd9) |
| 132.1 | Pediculus Corporis (Icd9) |
| 132.2 | Phthirus Pubis (Icd9) |
| 132.3 | Mixed Pedicul & Phthirus (Icd9) |
| 132.9 | Pediculosis Nos (Icd9) |
| B85.0 | Pediculosis Due To Pediculus Humanus Capitis (ICD10) |
| B85.1 | Pediculosis Due To Pediculus Humanus Corporis (ICD10) |
| B85.2 | Pediculosis, Unspecified (ICD10) |
| B85.3 | Phthiriasis (ICD10) |
| **ICD Code** | **Classification** |
| B85.4 | Mixed Pediculosis And Phthiriasis (ICD10) |
|  |  |
| **Other** | |
| 134.1 | Arthropod Infest Nec (Icd9) |
| 134.8 | Infestation Nec (Icd9) |
| 134.9 | Infestation Nos (Icd9) |
| B88.9 | Infestation (Skin) Nos, Infestation By Mites Nos, Skin Parasites Nos (Icd10) |
